# Comparison between dynamic gadoxetate-enhanced MRI and ^99m^Tc-mebrofenin hepatobiliary scintigraphy with SPECT for quantitative assessment of liver function

**DOI:** 10.1007/s00330-019-06029-7

**Published:** 2019-02-22

**Authors:** F. Rassam, T. Zhang, K. P. Cieslak, C. Lavini, J. Stoker, R. J. Bennink, T. M. van Gulik, L. J. van Vliet, J. H. Runge, F. M. Vos

**Affiliations:** 1 Department of Surgery, Cancer Center Amsterdam, Amsterdam UMC, University of Amsterdam, Amsterdam, The Netherlands; 2 Quantitative Imaging Group, Faculty of Applied Sciences, Delft University of Technology, Delft, The Netherlands; 3 Departments of Radiology and Nuclear Medicine, Cancer Center Amsterdam, Amsterdam UMC, University of Amsterdam, Amsterdam, The Netherlands

**Keywords:** Liver function tests, Technetium Tc 99m mebrofenin, Gadolinium ethoxybenzyl DTPA, Magnetic resonance imaging

## Abstract

**Objectives:**

To compare Gd-EOB-DTPA dynamic hepatocyte-specific contrast-enhanced MRI (DHCE-MRI) with ^99m^Tc-mebrofenin hepatobiliary scintigraphy (HBS) as quantitative liver function tests for the preoperative assessment of patients undergoing liver resection.

**Methods:**

Patients undergoing liver surgery and preoperative assessment of future remnant liver (FRL) function using ^99m^Tc-mebrofenin HBS were included. Patients underwent DHCE-MRI. Total liver uptake function was calculated for both modalities: mebrofenin uptake rate (MUR) and Ki respectively. The FRL was delineated with both SPECT-CT and MRI to calculate the functional share. Blood samples were taken to assess biochemical liver parameters.

**Results:**

A total of 20 patients were included. The HBS-derived MUR and the DHCE-MRI-derived mean Ki correlated strongly for both total and FRL function (Pearson *r* = 0.70, *p* = 0.001 and *r* = 0.89, *p* < 0.001 respectively). There was a strong agreement between the functional share determined with both modalities (ICC = 0.944, 95% CI 0.863–0.978, *n* = 20). There was a significant negative correlation between liver aminotransferases and bilirubin for both MUR and Ki.

**Conclusions:**

Assessment of liver function with DHCE-MRI is comparable with that of ^99m^Tc-mebrofenin HBS and has the potential to be combined with diagnostic MRI imaging. This can therefore provide a one-stop-shop modality for the preoperative assessment of patients undergoing liver surgery.

**Key Points:**

*• Quantitative assessment of liver function using hepatobiliary scintigraphy is performed in the preoperative assessment of patients undergoing liver surgery in order to prevent posthepatectomy liver failure.*

*• Gd-EOB-DTPA dynamic hepatocyte-specific contrast-enhanced MRI (DHCE-MRI) is an emerging method to quantify liver function and can serve as a potential alternative to hepatobiliary scintigraphy.*

*• Assessment of liver function with dynamic gadoxetate-enhanced MRI is comparable with that of hepatobiliary scintigraphy and has the potential to be combined with diagnostic MRI imaging.*

**Electronic supplementary material:**

The online version of this article (10.1007/s00330-019-06029-7) contains supplementary material, which is available to authorized users.

## Introduction

Surgical resection remains the only curative treatment in patients with primary and metastatic liver tumors and is presently performed with limited morbidity and mortality [1, 2]. However, extended liver resection still comes with the risk of posthepatectomy liver failure (PHLF) with incidence reported between 0.7 and 9.1% [3]. An insufficient future remnant liver (FRL) is one of the most important risk factors for the development of PHLF. The current management of PHLF is merely supportive and has a mortality rate of over 80% [4, 5]. Therefore, preoperative assessment liver function is crucial in order to minimize the risk of developing PHLF.

Several quantitative dynamic liver function tests are currently used to assess hepatic uptake and excretory function. This can be done with hepatobiliary scintigraphy (HBS) using technetium-99m (^99m^Tc)-labeled iminodiacetic acid derivates of which mebrofenin is the most hepatocyte specific [6]. This lidocaine analogue is taken up by the hepatocytes and is excreted in the bile canaliculi without undergoing any biotransformation [7]. The hepatic uptake is facilitated by the same mechanisms as other endo- and exogenous substances (e.g., bilirubin and hormones), making it a favorable agent to assess liver uptake and excretory function [8]. Because HBS provides a direct quantitative measure of the uptake function, it can be used in both patients with healthy or impaired liver parenchyma (e.g., steatosis, hepatitis, and fibrosis) using the same cutoff value for the uptake rate (2.7%/min/m^2^) [9]. Furthermore, HBS is combined with SPECT-CT which provides information on the regional distribution of liver function, enabling a more anatomical evaluation of FRL function [10].

HBS has proven to predict the risk of PHLF in a mixed series of patients undergoing major liver resection and is part of standard practice for the preoperative assessment of patients undergoing liver resection in our center [9, 11–13]. Even though HBS provides simultaneous morphologic (visual) and physiologic (functional) information of the liver, it is not suitable for diagnostic purposes due to the relatively low spatial resolution. Patients undergo additional imaging for diagnostic purposes.

Alternatively, MRI with gadolinium ethoxybenzyl diethylenetriaminepentaacetic acid (Gd-EOB-DTPA; Primovist®) as a contrast agent for evaluation of liver function was first performed in 1993 [14]. Subsequently, multiple studies showed correlation with liver function in both animal models and humans [15–22]. Gd-EOB-DTPA shares pharmacokinetic properties with mebrofenin, as both are taken up by hepatocytes and are excreted in the bile canaliculi without undergoing biotransformation. Furthermore, dynamic hepatocyte-specific contrast-enhanced MRI (DHCE-MRI) with gadolinium-based contrast agents allows accurate depiction of benign or malignant liver lesions [23–25]. Pharmacokinetic models have been developed that facilitate the estimation of the uptake rate of the contrast agent based on DHCE-MRI with Gd-EOB-DTPA on a *per voxel basis* [26–28]. Diagnostic MRI followed by DHCE-MRI therefore potentially provides a detailed, one-stop-shop modality for both diagnostic purposes as well as accurate determination of FRL function.

The aim of this study is to compare Gd-EOB-DTPA-enhanced DHCE-MRI with ^99m^Tc-mebrofenin HBS as liver function tests for the preoperative assessment of patients undergoing liver resection. We hypothesize that the liver function determined by DHCE-MRI correlates with ^99m^Tc-mebrofenin HBS.

## Materials and methods

### Patients

Patients diagnosed with one or more liver lesions and who were scheduled for ^99m^Tc-mebrofenin HBS as part of the preoperative workup were included in this prospective observational pilot study. Patients with general contraindications for MRI, chronic renal insufficiency, known or family history of congenital prolonged QT syndrome, current use or history of arrhythmia after the use of cardiac repolarization time-prolonging drugs, and allergy to gadolinium-containing compounds were excluded from participation. As this was a pilot study, no formal sample size calculation was performed. The study was approved by the ethical review board of the Amsterdam University Medical Centers and registered under ID NL45755.018.13. Informed consent was obtained from all individual participants included in the study.

### Hepatobiliary scintigraphy

**Fig. 1:**
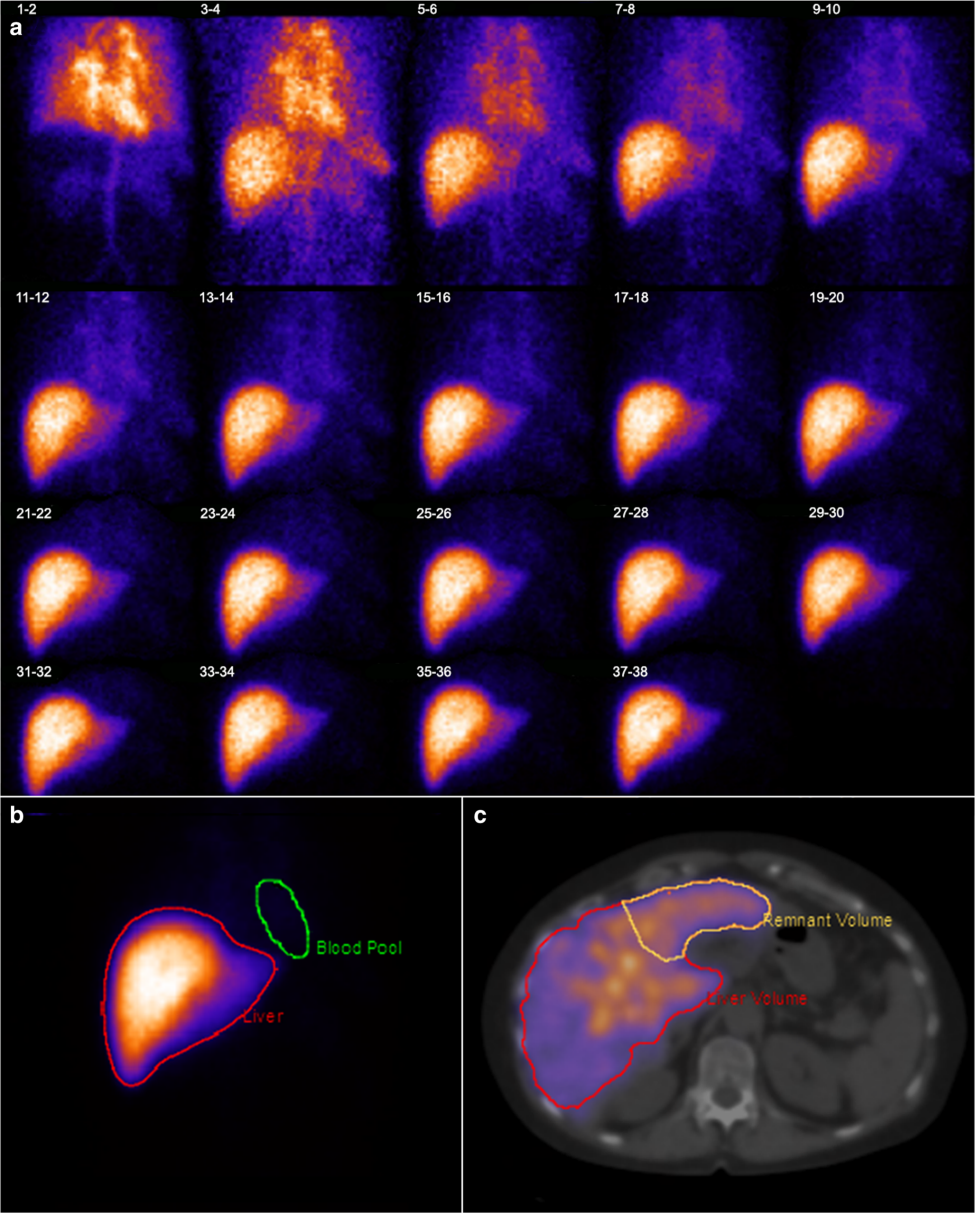
Hepatobiliary scintigraphy with series scintigram (**a**), ROI on summed images (**b**), and FRL delineation on SPECT/CT (**c**)

All patients underwent HBS to evaluate total and FRL function prior to resection as described previously [9, 10]. Briefly, dynamic acquisitions were obtained using a dual head SPECT-CT camera (Siemens Symbia T16) for 38 frames of 10 s/frame after injection of 200 MBq 99m Tc-mebrofenin (Bridatec, GE Healthcare) in order to calculate the hepatic uptake rate (Fig. 1a). Subsequently, SPECT was performed (60 projections of 8 s/projection, 128 matrix) which was used for the three-dimensional assessment of liver function and calculation of functional liver volume. This was combined with low-dose, non-contrast-enhanced CT for attenuation correction and anatomical mapping. Finally, dynamic acquisitions were obtained (15 frames; 60 s/frame, 128 matrix) to evaluate biliary excretion.

Data were processed on a Hermes workstation (Hermes Medical Solutions). Geometric mean datasets of the anterior and posterior acquisitions were used for the analysis [10]. Regions of interest (ROI) were drawn delineating the liver, the left ventricle and aorta (representing the blood pool), and the total field of view (FOV), from which time-activity curves were created (Fig. 1b).

Total liver function (TLF) was represented by the mebrofenin uptake rate (MUR; %/min). This was calculated as an increase of ^99m^Tc-mebrofenin uptake over a time period of 200 s as described by Ekman et al [29].

The FRL was defined on the planned resection and was delineated manually on the SPECT-CT images to calculate its functional share from HBS (FS HBS), which was defined as the fraction of counts within the FRL (Fig. 1c). Subsequently, this functional share fraction was multiplied by the TLF to calculate the FRL function (fMUR; %/min).

### DHCE-MRI

**Fig. 2:**
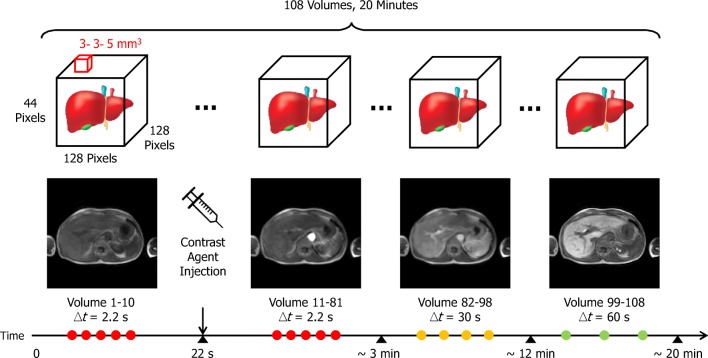
DHCE-MRI protocol

DHCE-MRI data were acquired on a 3.0 Philips Ingenia whole-body MR scanner (Philips Healthcare) by means of a dynamic T1-weighted 3D spoiled gradient echo sequence. The acquisition parameter settings were TE/TR = 2.30/3.75 ms, FA = 15°, matrix size = 128 × 128 × 44, voxel size = 3 × 3 × 5 mm^3^, acquisition time = 2.14 s for each volume; sampling interval (between images) was 2.14 s for volumes 1–81, 30 s for volumes 82–98, and 60 s for volumes 99–108. The total imaging for the dynamic series time was approximately 20 min. Subjects held their breath during the acquisition of volumes 13–22, 33–42, 61–70, and 79–108. Upon acquisition of dynamic 11 (i.e., 21 s after the start of the DHCE acquisition), a bolus of Gd-EOB-DTPA (Primovist®, Bayer B.V.) at a standard dose of 0.025 mmol/kg (i.e., 0.1 mL/kg) was administered at 2 mL/s and flushed with 20 mL of saline at the same rate through an antecubital intravenous cannula (Fig. 2).

All postprocessing was performed with in-house developed software implemented in MATLAB (R2015b; MathWorks). Details regarding the applied techniques are provided in ESM Appendix A.

In order to estimate the liver function, the pharmacokinetics of the liver was modeled from the MRI data based on Sourbron’s model [26]. This model yielded the Gd-EOB-DTPA uptake rate (min^−1^) in each voxel of the liver, which was averaged over the entire liver segmentation to represent the total liver’s uptake rate Ki as measured with DHCE-MRI.

**Fig. 3:**
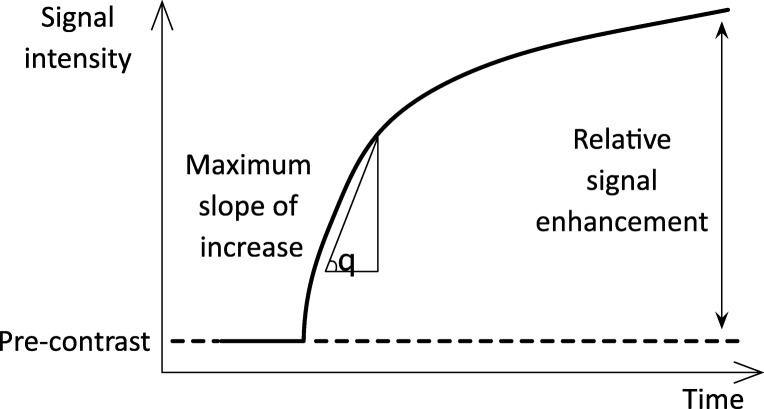
Signal intensity curve with semi-quantitative parameters

Additional semi-quantitative MRI study parameters were the relative enhancement (RE) and maximum slope of increase (MSI) (Fig. 3). RE was defined in each voxel as the difference of the signal at 20 min with the signal at baseline divided by the signal at baseline. MSI is defined as the maximum slope along the signal’s time course. Both parameters were averaged over the entire liver.

For the calculation of the functional share from MRI (FS MRI), the FRL was manually delineated in the last dynamic (showing the largest contrast) using an ROI drawing tool. FS MRI was calculated as the summed Ki values in the FRL divided by the sum of Ki values over all voxels of the liver. Additionally, the FRL function from MRI was calculated as mean Ki in the delineated FRL region (fKi). Similarly, the mean ME and MSI were calculated over the FRL region.

### Biochemical parameters

Blood samples were collected immediately before the MRI scan for routine laboratory evaluation of aspartate aminotransferase (AST), alanine aminotransferase (ALT), bilirubin, albumin, prothrombin time (PT), international normalized ratio (INR), and creatinine.

### Statistical analysis

Continuous data were summarized by median and interquartile range (IQR) if not normally distributed and as mean and standard deviation (SD) when normally distributed. Discrete variables were expressed as absolute numbers and relative frequencies. Pearson rank correlation was performed to analyze the relation between normally distributed variables. Reproducibility was assessed using intra-class correlation coefficient (absolute agreement, single measures, two-way mixed) and by a Bland-Altman plot. Statistical analysis was performed with IBM SPSS Statistics (version 24.0; IBM Corp).

## Results

### Patients

Between December 2014 and July 2018, 20 patients underwent DHCE-MRI within 2 weeks of the ^99m^Tc-mebrofenin HBS. Patient characteristics are presented in Table 1. The median (IQR) time between HBS and MRI was 5 (2–10) days.

**Table 1:** Patient characteristics

Characteristics, *n* = 20	
Age, median (IQR)	64 (57–70)
Male sex, *n* (%)	12 (67%)
BMI, kg/m^2^, median (IQR)	22.5 (21.3–28.2)
BSA, m^2^, median (IQR)	1.9 (1.7–2.1)
Diagnosis, *n* (%)	
Colorectal liver metastasis	9 (45%)
Biliary tumor	4 (20%)
Hepatocellular carcinoma	3 (15%)
Benign	4 (20%)
Neo-adjuvant chemotherapy, *n* (%)	6 (30%)
Preoperative biliary drainage, *n* (%)	3 (15%)
Histology non-tumorous parenchyma	
No histology	1 (5%)
Normal	17 (85%)
Fibrosis	1 (5%)
Cirrhosis	1 (5%)

### Liver function

**Fig. 4:**
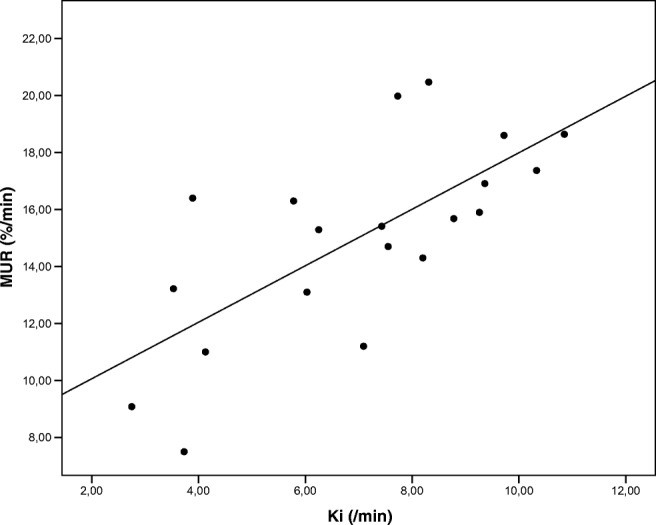
Pearson correlation between total liver function represented by the mebrofenin uptake rate (MUR; %/min) and the Gd-EOB-DTPA uptake rate (Ki; min^−1^)

The mean MUR for the total liver averaged over all patients was 15.1 (± 3.4) %/min. The mean Gd-EOB-DTPA uptake rate of the whole liver (Ki) averaged over all patients was 7.0 (± 2.4) per minute. There was a strong correlation between the MUR and Ki (Pearson *r* = 0.70, *p* = 0.001, *n* = 20) (Fig. 4).

### Functional share and future remnant liver function

**Fig. 5:**
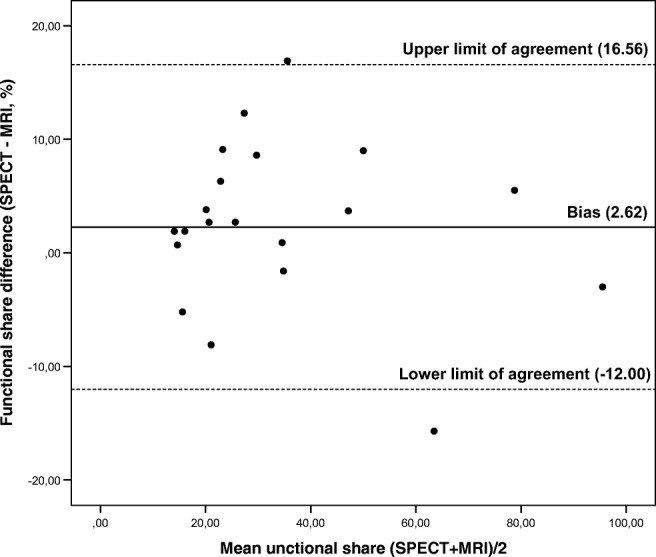
Bland-Altman plot for the agreement between functional share (%) of the FRL measured with SPECT and MRI

There was a strong agreement between the functional shares from HBS (FS HBS) and MRI (FS MRI) (ICC = 0.944, 95% CI 0.863–0.978, *n* = 20). A Bland-Altman plot is presented in Fig. 5. The mean difference in the functional share between FS HBS and FS MRI was 2.6% and the 95% limit of agreement was ± 14.3%.

**Fig. 6:**
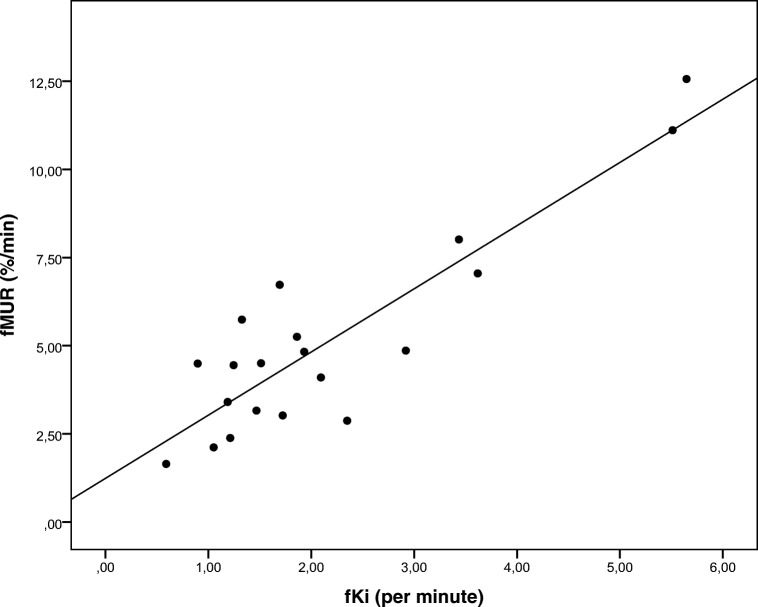
Pearson correlation between future remnant liver function represented by the mebrofenin uptake rate (fMUR; %/min) and the Gd-EOB-DTPA uptake rate (fKi; min^−1^)

Additionally, there was a strong correlation between the FRL function measured from HBS (fMUR) and MRI (fKi) (Pearson *r* = 0.89, *p* < 0.001, *n* = 20) (Fig. 6).

### Biochemical parameters

Blood samples were taken from all patients. In one patient, albumin and PT could not be determined and in three patients, INR could not be obtained, due to failing processing of the blood samples. Total serum bilirubin was marginally elevated in three patients (32, 34, and 42 μmol/L respectively). There was a negative correlation between AST, ALT, and bilirubin for both MUR and Ki (Table 2).

**Table 2:** Pearson correlation between blood parameters and MUR and Ki

		AST	ALT	Bilirubin	Albumin	PT	INR	Creatinine
MUR	*r*	0.656	− 0.530	− 0.776	0.224	*−* 0.144	*−* 0.217	*−* 0.325
	*p*	0.002**	0.002**	< 0.001**	0.356	0.557	0.403	0.161
	*n*	20	20	20	19	19	17	20
Ki	*r*	− 0.603	− 0.525	− 0.633	*−* 0.01	*−* 0.059	*−* 0.063	*−* 0.215
	*p*	0.005**	0.017*	0.003**	0.968	0.810	0.811	0.362
	*n*	20	20	20	19	19	17	20

*p < 0.05, **p < 0.01

### Semi-quantitative parameters

There was a moderate correlation between RE and the MUR (Pearson *r* = 0.473, *p* = 0.039, *n* = 20). Furthermore, there was no significant correlation between the mean MSI and the MUR (Pearson *r* = − 0.380, *p* = 0.098, *n* = 20).

## Discussion

In this study, we demonstrated that there was a strong correlation between liver function measured with the mebrofenin uptake rate (MUR) derived from ^99m^Tc-mebrofenin HBS and the mean Ki from DHCE-MRI in patients with planned liver resection. Furthermore, there was a strong agreement between the functional share of the FRL, measured with the SPECT-CT and MRI, yielding comparable calculations of the FRL function for both modalities. Sourbron’s model provided a quantification of the uptake rate of Gd-EOB-DTPA which is comparable to the MUR. To our knowledge, this is the first study to compare the functional distribution of liver function between HBS and MRI as such.

Geisel et al compared in an earlier study the liver function measured with HBS and MRI of the left and right liver lobes in patients undergoing portal vein embolization [30]. They showed a moderate to strong correlation between both the relative enhancement and the hepatic uptake index on MRI and mebrofenin uptake in HBS.

For the evaluation of liver function, we rely on the hepatic uptake of liver-specific agents. This uptake depends on liver perfusion, vascular permeability, extracellular diffusion, and hepatocyte transport, which parameters are taken into account in Sourbron’s model. Clearly, all these parameters can be altered during liver disease. We hypothesize that by explicitly taking them into account into Sourbron’s model, Ki yielded improved (strong) correlation with MUR over the semi-quantitative parameters. Specifically, we found only moderate correlation between RE and MUR while the correlation between MSI and MUR was not significant.

There are several similarities in pharmacokinetic properties between mebrofenin and Gd-EOB-DTPA, in particular, the uptake and excretion by the same transporters [31, 32]. Accordingly, multiple studies have shown that the liver enhancement effects of Gd-EOB-DPTA, which were (semi)-quantitatively assessed, depend on liver function [18, 33, 34]. The main difference between both substances is that mebrofenin is exclusively excreted by the liver, whereas approximately 50% of the injected Gd-EOB-DTPA dose is taken up by the hepatocytes and 50% is excreted through the renal system (assuming normal kidney function) [35]. In the absence of adequate biliary excretion, the urinary excretion pathway can compensate for any deficient hepatic transport mechanism [36, 37]. Renal excretion was found to be increased in patients with severe hepatic impairment. Even in that case, however, a high hepatic signal has been observed, which was adequate to quantitatively assess liver function [37].

Several pharmacokinetic models have been proposed to estimate liver function from DHCE-MRI. Nilsson et al applied a technique called truncated singular value decomposition in order to estimate pharmacokinetic properties [38]. However, this approach regarded the hepatic artery as the sole input and ignored the portal vein. Sourbron et al created a dual-input, two-compartmental model that accounted for Gd-EOB-DTPA metabolization by the hepatic cells in 2012 [26]. One limitation of this model is that it ignores the extraction rate of hepatocytes, i.e., the efflux to the bile canaliculi. To solve this, Ulloa et al and Forsgren et al modeled the transport of the contrast agent from the hepatocytes to the bile via so-called Michaelis-Menten kinetics in rats and humans, respectively [27, 39]. Alternatively, Georgiou et al modified the efflux transport component of this model by a simpler approximation [28]. Truhn et al developed a model that allows simultaneous quantification of gadoxetic acid uptake and excretion [40]. Recently, Ning et al correlated pharmacokinetic parameters estimated from different models with a blood chemistry test [41]. They report that the relative liver uptake rate estimated from the model without bile efflux transport correlated with direct bilirubin (*r* = − 0.52, *p* = 0.015), prealbumin (*r* = 0.58, *p* = 0.015), and PT (*r* = − 0.51, *p* = 0.026). Furthermore, only insignificant correlations were found using the model with efflux transport. For this reason, we applied Sourbron’s model in our work.

A variety of biochemical blood tests reflect the numerous functions of the liver. We focused on ALT and AST levels which reflect liver damage or hepatotoxicity, coagulation parameters like PT and INR, albumin that reflect synthesis function, and bilirubin which is generally considered the most potent prognostic marker for liver disease and has been used in numerous prognostic models [42, 43]. We found moderate to strong correlations between the hepatic uptake of both mebrofenin and Gd-EOB-DTPA and AST, ALT, and serum bilirubin, confirming earlier findings [44, 45].

During hyperbilirubinema, which is often the case in patients with obstructive biliary tumors, there is competitive uptake of mebrofenin/Gd-EOB-DTPA and bilirubin by the hepatocytes due to the binding to the same receptor. This could explain the strong negative correlations between plasma bilirubin and the uptake of both Gd-EOB-DTPA and mebrofenin in this cohort. Furthermore, during cholestasis, efflux of bile is impaired which can further contribute to the decreased Gd-EOB-DTPA uptake. It is therefore necessary that patients with hyperbilirubinemia should undergo adequate biliary drainage before undergoing either HBS or DHCE-MRI.

The absence of correlation between albumin and coagulation parameters might be explained by insufficient power (due to the small patient population) and the absence of patients with severely impaired liver function.

A disadvantage of HBS is (despite the relatively low radiation burden) the relatively low spatial resolution, making it not feasible as a diagnostic modality for the differentiation of liver lesions. On the contrary, the best available imaging tool for lesion characterization is standard contrast-enhanced MRI with multiple contrast phases. With conventional MR imaging techniques, DHCE-MRI cannot be combined with the standard contrast-enhanced scans; one performs either of the two scan types. New developments in the field of MRI sequence engineering now offer the possibility to acquire data continuously in free breathing using a radial acquisition scheme [46, 47]. When performed before and during contrast administration, the raw data can be reconstructed into different data sets: (1) the standard contrast phases optimally timed for each subject as the inflow of contrast-enhanced blood into the liver can be observed and (2) a dynamic contrast-enhanced data set for time-intensity curve and/or pharmacokinetic analysis. While this has not yet been evaluated, the application of such a radial acquisition in this patient group could provide a one-stop-shop modality where patients undergo one scan for both characterization of underlying liver disease and evaluation of liver function.

Furthermore, MRI facilitates evaluation of fibrosis, steatosis, and micro-perfusion levels of the hepatic tissue as well as assessment of bile duct disease [48–50]. These parameters were not measured in our cohort because most patients had relatively normal (global) liver function without great variation in fibrosis or steatosis grade. Future studies could focus on the relation between Gd-EOB-DTPA uptake and fibrosis or steatosis grade assessed with histopathological quantification of liver biopsies.

One limitation of the applied MRI protocol was that the patients were instructed to hold their breath at several time points. We did so to avoid movement artifacts during image acquisition, especially at the time points corresponding to the arterial, portal-venous, and late venous phases. A free-breathing DHCE sequence, for example, with a radial acquisition scheme, should be studied in the future in order to reduce the burden on patients.

Another limitation of this study is the rather small variation in liver function in our cohort. A larger variation would potentially yield an increase of the correlation between HBS and MRI. The small sample size might also result in insufficient power to detect a significant correlation with other blood samples like coagulation parameters. Future studies in a different study population including patients with chronic and diffuse liver disease that have a wider range of liver function should be conducted to make these findings more robust.

We did not perform an exact sample size calculation (a power analysis), since there was no previous data available on the correlation between Sourbron’s model parameters and MUR. We anticipate that our data can form the basis for sample size calculation for a larger prospective, observational cohort study.

In conclusion, assessment of liver function with DHCE-MRI is comparable with that of ^99m^Tc-mebrofenin HBS. If future studies confirm these findings and new free-breathing scan techniques can be applied successfully, DHCE-MRI could provide a one-stop-shop modality for the preoperative assessment of patients undergoing liver surgery.

## Electronic supplementary material

ESM 1 (DOCX 118 kb)

## Abbreviations

^99m^Tc: Technetium 99m
ALT: Alanine aminotransferase
AST: Aspartate aminotransferase
DHCE-MRI: Dynamic hepatocyte-specific contrast-enhanced MRI
fKi: Mean Ki in the future remnant liver
fMUR: Future remnant liver function
FOV: Field of view
FRL: Future remnant liver
FS HBS: Functional share from HBS
FS MRI: Functional share from MRI
Gd-EOB-DTPA: Gadolinium ethoxybenzyl diethylenetriaminepentaacetic acid
HBS: Hepatobiliary scintigraphy
INR: International normalized ratio
MSI: Maximum slope of increase
MUR: Mebrofenin uptake rate
PHLF: Posthepatectomy liver failure
PT: Prothrombin time
RE: Relative enhancement
TLF: Total liver function
