# Supplementary material for: Comparison between dynamic gadoxetate-enhanced MRI and 99mTc-mebrofenin hepatobiliary scintigraphy with SPECT for quantitative assessment of liver function
Source: Eur Radiol. 2019 Feb 22;29(9):5063–72. doi: 10.1007/s00330-019-06029-7 (PMC6682576; doi:10.1007/s00330-019-06029-7)
Supplement: Supplementary file 1 — (DOCX 118 kb) [file 330_2019_6029_MOESM1_ESM.docx]

**Appendix A: MRI image processing**

The dynamic images were aligned to the first image in the series using the Modality Independent Neighborhood Descriptor (MIND) method [1]. As such, spatial correspondence of the images was achieved across time. Subsequently, the liver was delineated using a semi-automatic segmentation method [2, 3]. The signal in each liver voxel was converted into time concentration curves assuming a nonlinear relationship between signal intensity and concentration of the contrast agent [4]. Furthermore, the liver’s arterial input function (AIF) and its portal venous input function (VIF) describing the contrast agent concentration in the blood plasma supplied to the liver by the hepatic artery and the portal vein were estimated by averaging the top three of most enhancing time concentration curves of the voxels from homogeneous regions in the aorta and the portal vein respectively [2].

The arterial input function *C*_A_ and venous input function *C*_V_ represent the dual inlets into the liver. *T*_A_ and *T*_V_ are time delays and *F*_A_ and *F*_V_ are constants representing the volume transfer rates from the plasma compartments into the extravascular, extracellular space. Furthermore, the gray rectangle denotes liver tissue, the left circle represents the extravascular extracellular compartment and the right circle stands for the extravascular intracellular compartment, i.e. corresponding to the hepatocytes. As such, *V*_E_ is the extravascular extracellular volume and *K*_I_ represents the uptake rate of the hepatocytes represented by a volume *V*_I_.

Figure A. Sourbron’s model : a dual-inlets, two-compartment uptake model for Gadoxetate disodium in the liver. The AIF and VIF are dual inlets into the liver, representing the concentration of the contrast agent over time entering from the hepatic artery and the portal vein. T_A_ and T_V_ are time delays. F_A_ and F_V_ are the arterial and venous plasma flows, respectively (mL/min/100 mL). The gray rectangle represents the liver, the left circle the extravascular extracellular compartment V_E_ (mL/100 mL) and the right circle stands for the hepatocytes, i.e. the extravascular intracellular compartment V_I_. K_I_ (per minute) is the liver uptake rate.

The analytical solution to Sourbron’s model is mathematically expressed as

$$\begin{matrix} C_{T}\left(t\right)=\frac{K_{I}}{F_{A}+F_{V}+K_{I}}\int_{0}^{t} \left[F_{A}C_{A}\left(\tau-T_{A}\right)+F_{V}C_{V}\left(\tau-T_{V}\right)\right]d\tau\\ +\frac{F_{A}+F_{V}}{F_{A}+F_{V}+K_{I}}e^{-\frac{t}{T_{E}}}\int_{0}^{t} e^{\frac{\tau}{T_{E}}}\left[F_{A}C_{A}\left(\tau-T_{A}\right)+F_{V}C_{V}\left(\tau-T_{V}\right)\right]d\tau, \end{matrix}$$

in which is the contrast agent concentration in a voxel, and

$$T_{E}=\frac{V_{E}}{F_{A}+F_{V}+K_{I}}.$$

This tracer kinetic model was fitted to the measured concentration in each voxel from the MRI scan using nonlinear least-squares fitting routine *lsqcurvefit* in MATLAB (version R2015b; Mathworks); 19 cores were adopted for parallel computing on a HPC equipped with two Intel(R) Xeon(R) CPU E5-2698 v4 clocked at 2.20GHz and 256GB RAM memory.

1. Heinrich MP, Jenkinson M, Bhushan M, et al. MIND: modality independent neighbourhood descriptor for multi-modal deformable registration. Med Image Anal. 2012;16(7):1423-35.

2. Zhang T, Li Z, Runge JH, et al. Improved registration of DCE-MR images of the liver using a prior segmentation of the region of interest. Medical Imaging 2016: Image Processing: SPIE; 2016.

3. Zhang Y, Matuszewski BJ, Shark L-K, Moore CJ**.** Medical Image Segmentation Using New Hybrid Level-Set Method. 2008 Fifth International Conference BioMedical Visualization: Information Visualization in Medical and Biomedical Informatics: IEEE; 2008:71-6.

4. Aronhime S, Calcagno C, Jajamovich GH, et al. DCE-MRI of the liver: effect of linear and nonlinear conversions on hepatic perfusion quantification and reproducibility. J Magn Reson Imaging. 2014;40(1):90-8.
